# Supplementary material for: Expression profile analysis of cotton fiber secondary cell wall thickening stage
Source: PeerJ. 2024 Jul 8;12:e17682. doi: 10.7717/peerj.17682 (PMC11238726; doi:10.7717/peerj.17682)
Supplement: Supplemental Information 1 [file peerj-12-17682-s001.docx]

**TABLE S1.** List of forward and reverse primers used for this study.

| Primer | Sequence (5'-3') | Purpose |
| --- | --- | --- |
| Polyphenol oxidase-RT-F | GAGGAAATCGAGGAGCAAAACTG | RT-qPCR |
| Polyphenol oxidase-RT-R | GCACCAAAGTCACCACAATACCAT | RT-qPCR |
| 3-ketoacyl-CoA synthase 3-RT-F | AATGGGGTGTAGTGCGAGTTTG | RT-qPCR |
| 3-ketoacyl-CoA synthase 3-RT-R | TAAAATCCCACCTTTCCTATCTCAT | RT-qPCR |
| 3-ketoacyl-CoA synthase 4-RT-F | AATCTTGGGGGTATGGGATGTAG | RT-qPCR |
| 3-ketoacyl-CoA synthase 4-RT-R | GCTTATACTTACTCCGCCTCCGAT | RT-qPCR |
| 3-hydroxyacyl-CoA dehydratase-RT-F | TGGCATCAGCAGTGAAGTTGGT | RT-qPCR |
| 3-hydroxyacyl-CoA dehydratase-RT-R | CCGAGCATATAACGGTACAAGTGAG | RT-qPCR |
| NAD(P)-binding Rossmann-fold superfamily protein-RT-F | ATCGGATCGGTTCATACTAAGCC | RT-qPCR |
| NAD(P)-binding Rossmann-fold superfamily protein-RT-R | AACATCTCCGCCAAAAGTAACAG | RT-qPCR |
| GDSL-like Lipase/Acylhydrolase superfamily protein-RT-F | TCGAAACCGGAATAAAGACACTGT | RT-qPCR |
| GDSL-like Lipase/Acylhydrolase superfamily protein-RT-R | ATTTTTTACAGAGGGTATGAAGTTGC | RT-qPCR |
| Caffeic acid O-methyltransferase 1-RT-F | CATTCTTCCTGATTACCCCGAC | RT-qPCR |
| Caffeic acid O-methyltransferase 1-RT-R | TGCCAAAAGCGGAACATGTAAT | RT-qPCR |
| WRKY transcription factor-RT-F | CCTTTTTGTTCAAGTCTGGGTCATA | RT-qPCR |
| WRKY transcription factor-RT-R | AAATGAGGTTTACCAGAACAAGGGA | RT-qPCR |
| 2,4-dienoyl-CoA reductase-RT-F | ACTCAATATCACCTGGGCTTTTCA | RT-qPCR |
| 2,4-dienoyl-CoA reductase-RT-R | ATGGGAACACCGGGTAAGGTAG | RT-qPCR |
| WRKY transcription factor 10-RT-F | CGGTAGTAAACCAGGCTTTGAATC | RT-qPCR |
| WRKY transcription factor 10-RT-R | GAATCTCGGCTCTCTCTGACTCTTA | RT-qPCR |
| Fatty acid desaturase 6-RT-F | TTATTGGAAGACATTGTGGGAACTC | RT-qPCR |
| Fatty acid desaturase 6-RT-R | AAATCAAAGTGCCATATCAACCAAT | RT-qPCR |
| GhHIS3-RT-F | GAAGCCTCATCGATACCGTC | RT-qPCR |
| GhHIS3-RT-R | CTACCACTACCATCATGG | RT-qPCR |
