# Supplementary figures and images for: Expression profile analysis of cotton fiber secondary cell wall thickening stage
Source: PeerJ. 2024 Jul 8;12:e17682. doi: 10.7717/peerj.17682 (PMC11238726; doi:10.7717/peerj.17682)

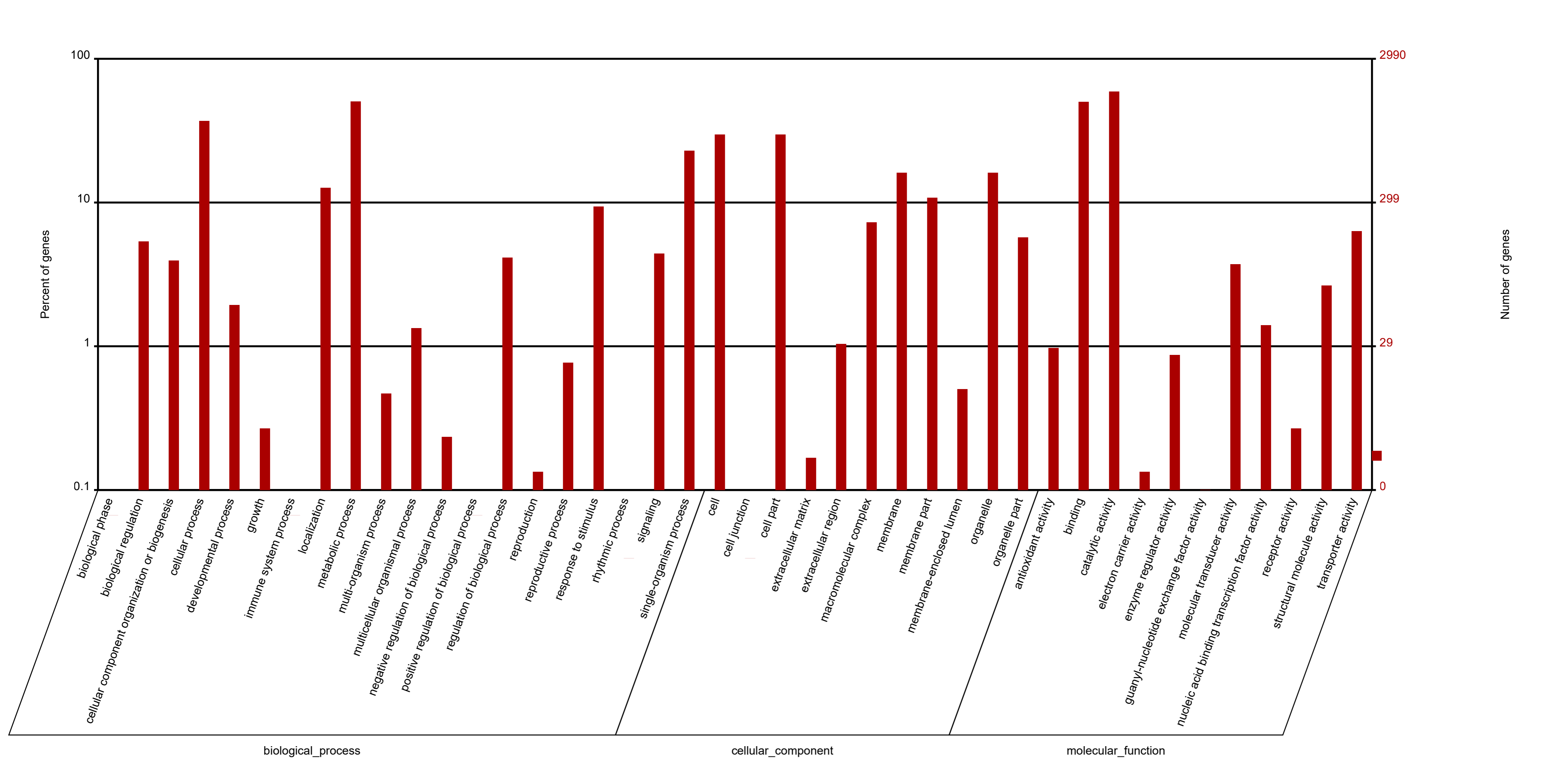

Supplement: Supplemental Information 2 — The X-axis represents the biological functions (molecular function, biological process, and cellular component) of these DEGs. The Y-axis represents the percentage or number of genes categorized into different functional pathways. [file peerj-12-17682-s002.pdf]

HL20-HL25-HL30

L20-L25-L30

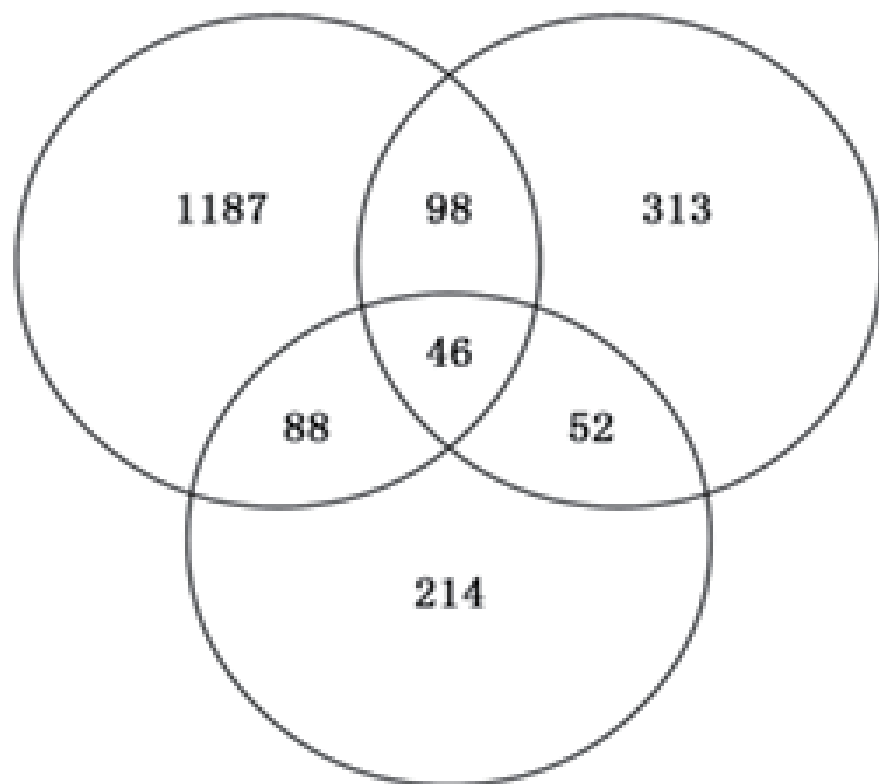

S20-L25-L30

Supplement: Supplemental Information 3 [file peerj-12-17682-s003.pdf]

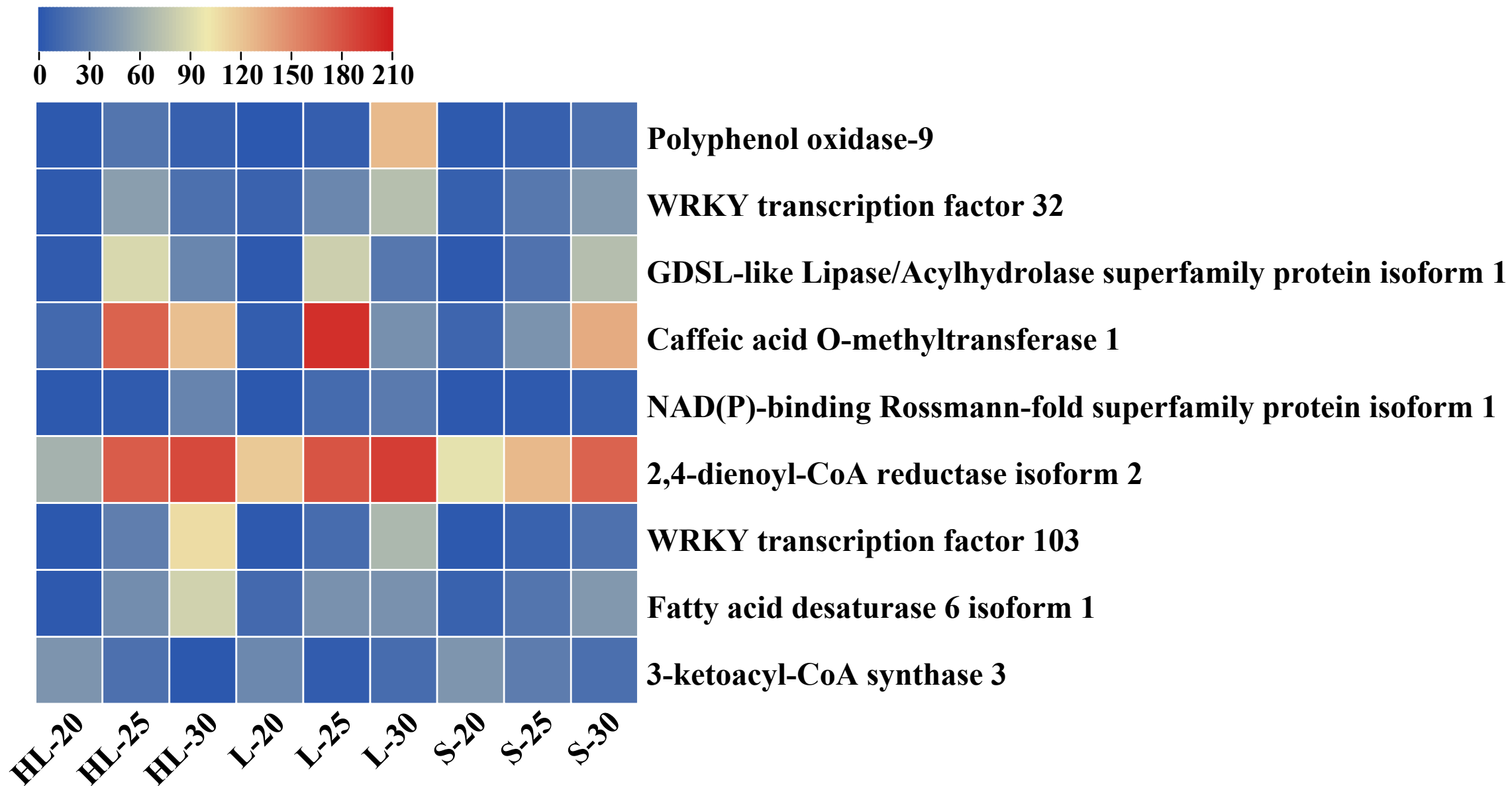

Supplement: Supplemental Information 7 — Note: HL represents Xinhai 32 (Sea Island cotton), L represents 17-24 (Upland cotton), and S represents 62-33 (Upland cotton). [file peerj-12-17682-s007.pdf]
