# Supplementary material for: Expression profile analysis of cotton fiber secondary cell wall thickening stage
Source: PeerJ. 2024 Jul 8;12:e17682. doi: 10.7717/peerj.17682 (PMC11238726; doi:10.7717/peerj.17682)
Supplement: Supplemental Information 6 [file peerj-12-17682-s006.docx]

Table S2. The correlation coefficient between the RT-qPCR and RNA-seq data of nine genes.

| GeneID-RNA-seq | Correlation coefficient |
| --- | --- |
| WRKY transcription factor 103 | 0.9989 |
| Polyphenol oxidase-9 | 0.9997 |
| WRKY transcription factor 32 | 0.9569 |
| GDSL-like Lipase/Acylhydrolase superfamily protein isoform 1 | 0.8747 |
| NAD(P)-binding Rossmann-fold superfamily protein isoform 1 | 0.8562 |
| 2,4-dienoyl-CoA reductase isoform 2 | 0.9905 |
| 3-ketoacyl-CoA synthase 3 | 0.9841 |
| Caffeic acid O-methyltransferase 1 | 0.9718 |
| Fatty acid desaturase 6 isoform 1 | 0.9701 |
